# Nanosecond multipulse retinal damage thresholds of elongated irradiance profiles in explant measurements and simulations

**DOI:** 10.1117/1.JBO.28.12.125001

**Published:** 2023-12-02

**Authors:** Marc Herbst, Sebastian Kotzur, Annette Frederiksen, Wilhelm Stork

**Affiliations:** a Cross-Domain Computing Solutions, Robert Bosch GmbH, Schwieberdingen, Germany; b Karlsruhe Institute of Technology, Institute for Information Processing Technologies, Karlsruhe, Germany; c University of Tübingen, Institute for Ophthalmic Research, Tübingen, Germany

**Keywords:** laser and eye safety, IEC 60825-1, laser induced retinal damage, multipulse retinal damage experiments, thermal simulation, laser tissue interaction

## Abstract

**Significance:**

The database for multipulse retinal damage thresholds for the laser safety standard (IEC 60825-1:2014) is confined, especially for elongated irradiation profiles. To ensure eye safety, retinal damage thresholds (${\mathrm{ED}}_{50}$ values) need to be determined.

**Aim:**

This study aims to examine nanosecond multipulse scenarios.

**Approach:**

To determine ${\mathrm{ED}}_{50}$ values in *ex vivo* measurements, an optical laser setup is presented. Porcine explant tissue is irradiated with rectangular top-hat profiles. Thermal simulations are carried out on a validated computer model and retinal injury thresholds are obtained.

**Results:**

The measurements resulted in ${\mathrm{ED}}_{50}$ values from 8.46 to 42.72  μJ with a slope from 1.15 to 1.4. A thermal damage in the measurements can be excluded due to the level value in combination with a different type of declining behavior for increasing pulses compared to the simulations. A dependence with increasing elongation or area of the retinal image emerges in the simulations but could not be confirmed in the measurements due to the influencing factors (biological variability, focusing, and measuring procedure).

**Conclusions:**

Using slit apertures for beam shaping, variable rectangular spot geometries are realized without changing elements in the setup. For further evaluation of the behavior of elongated irradiation profiles, additional measurements to improve the measurement accuracy are necessary.

## 1 Introduction

The manufacturers of laser systems are obliged to ensure the eye safety of their products. When using lasers in the wavelength range of visible- and near-infrared light, there is a risk of retinal damage if emission limits (EL) are exceeded. Therefore, scanning laser systems or laser optical systems have to fulfill national product safety standards (e.g., DIN EN 60825-1:2014^1^) for safe use. National laser safety standards are mostly derived by the international laser safety standard (IEC 60825-1:2014)^2^ and based on the guidelines of the International Commission on Non-Ionizing Radiation Protection (ICNIRP).^3^ The ICNIRP, as a committee of experts in the field of nonionizing radiation, reviews the existing database of experimental retinal threshold experiments and simulations and proposes guidelines on limits of exposure to laser radiation.^3^^–^^5^

**Fig. 1:**
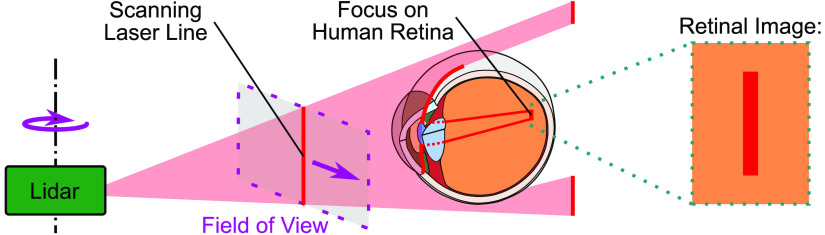
Optical scanning systems emit laser beams. The focusing of these laser beams on the retina of the human eye causes a potential risk of retinal damage. In the special case presented a line scanning, lidar sensor emits a divergent beam in the shape of a line, which results in an elongated, nonuniform irradiance profile on the retina (retinal image).

There are many applications of laser systems, in which direct exposure to the human eye has to be considered, e.g., range finding solutions, such as lidar systems, optical communication and scanning principles, and projectors. Laser radiation in the “retinal hazard region”^6^^,^^7^ (400 to 1400 nm) enters the human eye and the anatomical structure of the eye images the radiation on the retina (Fig. 1).^7^^–^^9^ The accommodation of the eye can lead to extreme cases, in which the laser radiation propagating into the eye concentrates on a small retinal area, resulting in high irradiance levels in the tissue.^10^ Excessively high irradiance levels lead to alteration of the tissue more specifically retinal injury due to photomechanical, thermomechanical, thermal, or photochemical damage mechanisms.^7^^,^^11^ Which one of these damage mechanisms is dominant depends primarily on the wavelength of the laser radiation, the exposure time,^7^ and the maximum optical power of the radiation.

In the past, researchers have created a substantial database of dose–response data by performing retinal damage threshold *in vivo* experiments, mostly on nonhuman primates (NHP).^8^ In spite of that, the “relevant experiments and their interpretations are problematic, and the evolutions of experimental techniques have generated an assortment of datasets that are difficult to reconcile.”^10^ Even though the possibility to perform *ex vivo* experiments with animal tissue has been used to expand the database in the past, there are still open questions on the significance of the individual experiments in terms of transferability and on the retinal damage mechanisms itself. This is the motivation for performing further experiments and expanding the database for laser safety.

To calculate the EL of laser systems and to classify laser products in general, manufacturers apply the laser safety standard IEC 60825-1:2014.^2^ If special characteristics of the laser systems are present due to a complex architecture of the systems, simplifications are made for the calculations. One characteristic is the nonuniformity of retinal spot geometries, which can be a special characteristic of scanning architectures, like lidar systems. Lidar systems use multipulsed laser sources and the resulting spot geometry or retinal images of these systems are usually not symmetrical or circular but rather nonuniform or elongated (Sec. 2 and Fig. 1). The laser safety standard provides guidance for nonuniform or elongated retinal images by including an averaging step. In this case, the averaging step for the nonuniform retinal spot size may result in a change of the area considered for the calculations compared to the present irradiation scenario (Sec. 2).

**Fig. 2:**
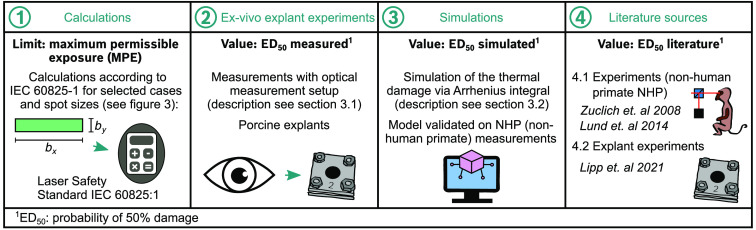
Overview of four major data sources of this work and their corresponding values defining a retinal damage or a limit for laser safety. In this work, data on behalf of the (1) laser safety standard (IEC 60825:1-2014), (2) new measurement data from *ex vivo* explant measurements, and (3) new data from thermal simulations are created. (4) Literature sources with experimental data collected with comparable measurement parameters are used as references.

In this work, retinal damage thresholds of nonuniform retinal spot geometries are determined in thermal simulations and *ex vivo* explant experiments using an optical setup with variable rectangular spot geometries (Sec. 3.1). The focus is on thermal simulations and the modeling of the thermal damage mechanism based on the Arrhenius integral, as this is currently the only established and validated approach of all damage mechanisms.^12^^–^^14^ In this work, three different types of data are generated and literature data is referenced. An overview of the four different data sources created and used in this work and the corresponding thresholds or values defining the retinal damage are presented in Fig. 2.

## 2 Elongated/Nonuniform Retinal Images and Their Calculation in the IEC 60825-1:2014

In laser products with scanning principles, e.g., lidar systems, micro or mechanical mirrors or other optical components are used to deflect the laser beam into a desired field of view. Due to the deflection and the beam shaping in the transmitting path of scanning lidar systems, the resulting beam profiles are not circular but elliptical or elongated or special shapes are created on purpose. Scanning lidar systems can be classified into MEMS lidar systems (biaxial or coaxial), mechanical lidar systems (point scan), and mechanical lidar systems (vertical line-scan).^15^^–^^19^ This work focuses on line-scan lidar systems, in which the usage of semiconductor lasers (e.g., vertical cavity surface emitting diodes arrays^15^^,^^17^) and the beam shaping within the transmitting path and the scanning unit can lead to a beam shape of a vertical divergent line that is almost collimated in the horizontal direction [e.g., “Valeo Scala (Gen 2) presented in Yole Market Report 2022^15^ or other patents^20^^–^^22^].

**Fig. 3:**
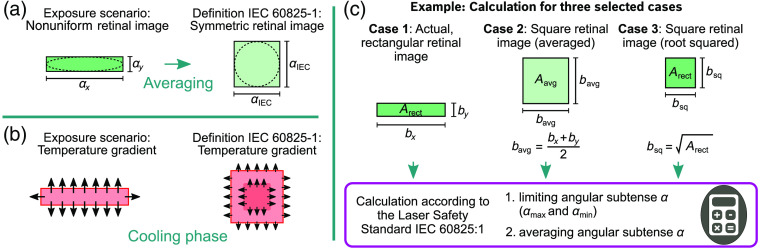
To calculate the EL according to the IEC 60825-1:2014, a symmetrization from nonuniform retinal images to symmetrical must be considered. (a) Therefore, the angular subtense $\alpha_{\mathrm{IEC}}$ has to be averaged from the values of the two dimensions $\alpha_{x}$ and $\alpha_{y}$ of the rectangular and elliptical retinal spot geometries. This may result in a change of the area considered for the calculation in comparison to the irradiated area. (b) The illustration of the cooling phase of a nonuniform versus the a symmetrical retinal image, as considered in the IEC 60825-1, shows that the temperature gradient of nonuniform irradiance profiles is higher, because of the elongated geometry. (c) Overview of all three selected cases used for performing exemplary eye safety evaluations of retinal images.

For the eye safety calculations of line-scan lidar systems, it is important to consider the exposure scenario, which includes the beam profile and can be a line or a rather strongly elongated elliptical shape approaching a line. Therefore, the resulting retinal images of the beam profiles are reviewed. In this work, the focus is on the steady-state retinal image of a line-scan lidar system with a geometry of a vertical line, not considering pupil-sweeps, which is a simplification and a first step of research. Divergent lines in general lead to elongated, nonuniform retinal spot geometries on the retina, which are considered in the IEC 60825-1:2014.^23^^–^^27^ For this purpose, the laser safety standard defines the angular subtense of the apparent source α, which is “the angle subtended by an apparent source viewed from a point in space.”^2^ The size of the retinal image is defined by the angular subtense α. For the calculation of damage thresholds of nonsymmetric retinal spot geometries, the laser safety standard IEC 60825-1:2014^2^ uses a step that simplifies the calculation. In this step, the arithmetic mean value, which is defined as $\alpha_{\mathrm{IEC}}$ in this work, of the two angular dimensions $\alpha_{x}$ and $\alpha_{y}$ has to be calculated [Fig. 3(a)].

The calculation of the mean value of the angular subtense $\alpha_{\mathrm{IEC}}$ (averaging step) has an impact on the considered area for the calculations and the cooling behavior. Some differences can be observed between elongated, nonuniform compared to symmetric (square or circular) spot geometries within the application of the IEC 60825-1:2014 and are illustrated in Figs. 3(a) and 3(b).

• *Considered area and irradiance*. In the laser safety standard, the area considered for the calculation may increase in comparison to the scenario of the elongated spot profile on the tissue (Fig. 3). A larger area can withstand a higher pulse energy without a retinal damage.^10^^,^^28^ In other words, the energy density may decrease in the calculation considered in the laser safety standard compared to the scenario of the elongated spot profile.
• *Cooling behavior to the side into the peripheral areas of the tissue*. The perimeter of the irradiated area does not change. The area or the volume of the tissue, where the heat flow can take place, remains the same. However, the temperature gradients in the cooling phase of centrally located partial areas are smaller in the scenario considered in the calculation [represented by smaller arrows in Fig. 3(b)]. Centrally located partial areas can cool down less quickly. (Assuming they would have been heated to the same temperature, which is not completely true, since the considered area of both scenarios may vary.)

Before the averaging step, the laser standard imposes a limitation of the angular subtense α [both $\alpha_{\max}$ and $\alpha_{\min}$; Fig. 3(c)]. Even with this limitation, there may be cases for nonuniform retinal images, where the area under consideration changes.

**Fig. 4:**
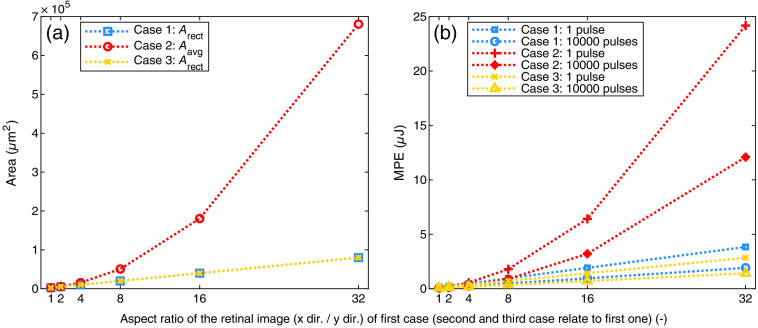
(a), (b) Results of the calculation example of three cases (Fig. 3) (irradiated area: $A_{\mathrm{rect}}$ and $A_{\mathrm{avg}}$) according to the laser safety standard.^2^ Parameters for the calculations are: wavelength 532 nm; pulse duration 2.2 ns; and pulse repetition rate 1000 Hz. The area $A_{\mathrm{avg}}$ of the second case increases along with the aspect ratio of the corresponding retinal image of case 1. The MPE value of all cases increases with increasing area of the retinal image. At higher pulse numbers, the calculated MPE value is lower, because the risk of retinal damage is increased.

To illustrate the effects of the averaging step described above, the maximum permissible exposure (MPE) values of ocular exposures of rectangular retinal images in the “retinal hazard region”^6^^,^^7^ are calculated on behalf of the laser safety standard^2^ (parameters in Fig. 4). In the calculation example, a distinction is made between three different selected cases [Fig. 3(c)]. In the first case, actual, rectangular, retinal images with the edge lengths $b_{y}=50\;\mu m$ and $b_{x}=50\;\mu m$ up to $b_{x}=1600\;\mu m$ (square to elongated) up to an aspect ratio of 1:32 are considered. The corresponding second and third cases have square retinal images, which are derived from the first case as described in Fig. 3(c). All retinal images are assigned to the respective retinal image of the first case, from which they are calculated. The first and the third cases share the same area $A_{\mathrm{rect}}$ of the actual retinal image, whereas the second case has a larger area to illustrate what happens to the irradiated area due to an averaging process. To calculate the MPE, the angular subtense $\alpha_{\mathrm{th}}$ used for the calculation of the threshold is calculated with the following equation:^29^

$$\alpha_{\mathrm{th}}=2\cdot \arctan(\frac{b}{2\cdot 17\cdot 10^{-3}}),$$ (1)

where b is the edge length of the irradiation profile or dimension of corresponding retinal image.

The results of the calculation example (Fig. 4) show the higher the aspect ratio or in other words the larger the elongation of the retinal image is, the larger are the differences of the MPE values and the area $A_{\mathrm{avg}}$ compared to $A_{\mathrm{rect}}$. This applies to both single and multipulse scenarios. Therefore, the calculation rules of the laser safety standard result in a higher MPE value in the first case than in the third case, even though both retinal images have the same area and thus the same irradiance level. So if an MPE value of an elongated retinal image is calculated (case 1), the received MPE value may be higher and more energy is applied, compared to the MPE value of an equivalent case with the same area (case 3). This implies a greater potential for retinal damage in the first case, which is why experimental and simulative data on elongated retinal images is needed, because the database contains, with the exception of scanning or moving symmetrical exposures (see Ref. 30 or Ref. 31), only damage measurements with symmetrical exposure scenarios so far.

## 3 Methods to Determine Retinal Thresholds

### 3.1 *Ex Vivo* Explant Experiments

#### 3.1.1 Samples and sample preparation

**Fig. 5:**
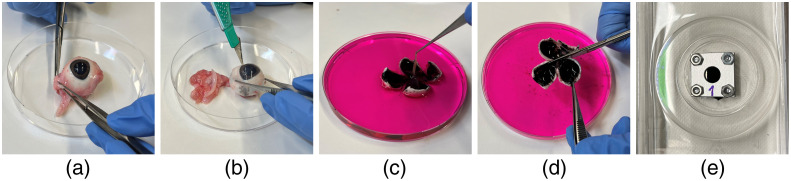
Preparation of the explant samples. (a) Preparation of the eyes in a large cell culture dish to remove the excess tissue. (b) Circular opening of the eyeball to remove the vitreous body and the anterior eye and cutting the eye bulbus into a shape of a cloverleaf. (c) Floating preparation of the sample; remove sensory part of the retina. (d) Cutting out samples of the cloverleaf and (e) fixation of the samples inside a sample holder.

Porcine eyes from three local slaughterhouses (Albmetzgerei Steinhard, Gammertingen, Germany; Emil Färber GmbH & Co. KG, Balingen, Germany; Emil Färber GmbH & Co. KG, Mengen, Germany) are used as explant tissue. Cooling of the samples is ensured until the preparation for the experiments. The preparation includes a multistep process (Fig. 5), where in the beginning, the excess tissue around the sclera, the lens and the anterior eye, vitreous body, and sensory retina is removed with medical cutlery, such as scissors and tweezers, and the eye bulbus is opened with an equatorial cut with a scalpel (Fig. 5), as introduced by Lipp et al.^32^^,^^33^

Histologically, the sample then consists of the retinal pigment epithelium (RPE), the bruchs membrane, the choroidea, and the underlying sclera and is fixated in a sample holder. The retinal area used as samples for irradiation is cut out of the sides of the eye bulbus so that the blind spot is not part of the sample. The entire time during the process, the sample, or respectively, the RPE cells are held alive, covered, and humidified with media or buffered saline solution, called Hanks’ Balanced Salt Solution (HBSS) (Carl Roth GmbH & Co. KG, Karlsruhe, Germany), or the staining solution mentioned in the following.

#### 3.1.2 Irradiation process with optical setup and ns-pulsed solid-state laser

**Fig. 6:**
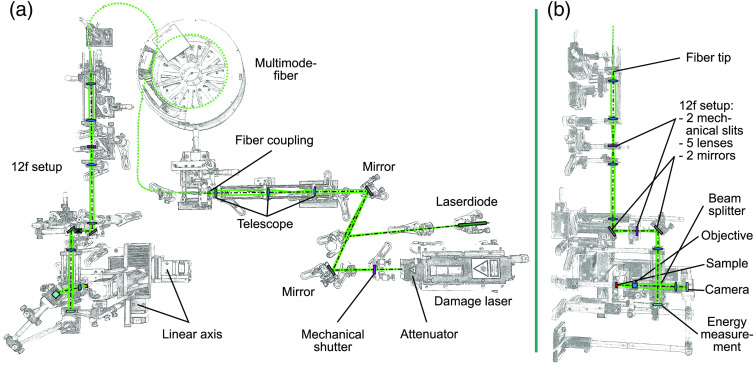
Optical setup for the irradiation of the samples. (a) The top view shows the table setup with the propagation of the laser beam colored in green. (b) The side view shows the beam shaping with a 12f setup, where the fiber tip is imaged on the sample plane resulting in a constant irradiance profile and variable rectangular spot geometries.

In this work, an optical setup (Fig. 6) to irradiate samples with variable rectangular spot geometries is presented. For the irradiation process, the samples are placed in a container and fully covered with the transparent HBSS [Fig. 5(e)]. The extinction of this saline solution is measured by the manufacturer in a comparative measurement with sterile water as a reference (sterile water, extinction reference: 0, transmission reference: 100; HBSS, extinction: 0.0007, and transmission: 100.06; 532 nm). For a propagation length of about 5 to 6 mm inside HBSS, it is therefore assumed that an attenuation of the pulse energy can be neglected for the measurements because the extinction is very low.

The optical setup (Fig. 6) is used to provide a marker on the sample in the shape of an “L” for orientation and as a reference for the irradiation procedure. Different numbers of measuring points up to 49 per sample with varying pulse energy and number of pulses (Table 2) are irradiated and automatically arranged in rows by a linear axis automatically controlled by a measuring software. In this optical setup, the q-switched nanosecond pulsed solid-state laser “FDSS 532-1000” (CryLaS Crystal Laser Systems GmbH, Berlin, Germany) is used to irradiate the sample. The laser is aligned with the laser diode “CPS532” (Thorlabs, Inc., Newton, New Jersey, United States; abbreviated as “Thorlabs” in the following for simplification purposes) for adjustment via one mirror “PF10-03-P01” and two mirrors “NB1-K12” (Thorlabs). The pulse duration of the laser is measured with the calibrated oscilloscope “HDO9404-MS” (Teledyne LeCroy, Chestnut Ridge, New York, United States) and the free-space photodetector “DET025AL/M” (Thorlabs) to 2.2 ns mean (Table 1) in the sample plane. The results show a “temporal broadening”^34^ of the pulses due to the propagation through the optical setup with included fiber, which is why a difference to the manufacturer’s specification of 1.7 ns in the sample plane results. The peak wavelength of the frequency doubled Nd:YAG laser is 532.46 nm (Table 1), measured by a calibrated spectrometer of the model type “Flame S” (Ocean Insight; Orlando, Florida, United States).

**Table 1:** Measurement results of the temporal (pulse duration) and spectral (wavelength) characteristics of the ns-pulsed laser in sample and measuring plane.

Measurement (temporal)	Value	Measurement (spectral)	Value
Pulse duration mean (FWHM^a^)	2.233 ns	Peak wavelength	532.46 nm
Pulse duration minimum (FWHM^a^)	1.957 ns	Center wavelength (fit)	532.18 nm
Pulse duration maximum (FWHM^a^)	2.347 ns	FWHM^a^ (fit)	2.52 nm
Standard deviation (FWHM^a^)	39.26 ps	FWQM^b^ (fit)	3.02 nm
Number of detected pulses	2169	Number of detections	100

a FWHM, full-width half maximum.

b FWQM, full-width quarter maximum.

**Table 2:** Irradiation scenarios of the explant experiments, number of pulses applied to the tissue per measurement point and cutting off of pulses to minimize the fluctuations of the pulse energy due to transient processes of the laser.

Scenario	Applied pulses	Duration (pulse package)	Cut off pulses	Duration (s) (cut off)
Single pulse	1	2.23 ns	5	0.2
Multipulse	100	5.0 s	40	1.95
Multipulse^a^	1000^a^	50 s^a^	40^a^	1.95^a^

a Only 280  μm×70  μm spot size.

The laser emits a Gaussian beam profile with ${\mathrm{TEM}}_{00}$ mode when triggered by a signal from the pulse generator “9514” (Quantum Composers Inc., Bozeman, Montana, United States). A pulse repetition rate of 20 Hz is used. Using an attenuator (CryLaS Crystal Laser Systems GmbH, Berlin, Germany), which is attached on the outlet of the laser, the pulse energy can be varied during the experiments and set to a defined value within the internal software. The laser is optimized for continuous pulsing, so this model’s pulse energy initially decreases and approximates the target value. To ensure a consistent pulse energy of every measurement point on the sample, the mechanical shutter “SHB025T” (Thorlabs) is used to cut off the first pulses of every measurement point (Table 2).

**Fig. 7:**
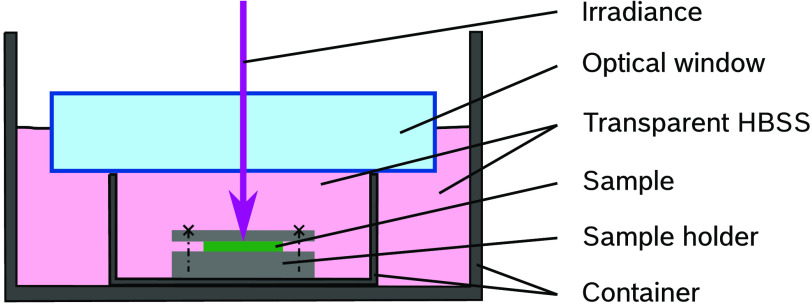
Irradiation of the sample moistened with HBSS to minimize oxidative stress and avoid dehydration (see Ref. 32). To improve the irradiation process, an optical window is used for the optical transition from air to the solution.

The Gaussian beam is coupled into a fiber by an optical telescope setup consisting of the three plano convex lenses “LA1027-A-ML,” “LA1509-A-ML,” and “LA1951-A” (Thorlabs). The multimode optical fiber “FP150QMT-CUSTOM” (Thorlabs) has a square core with a width of 150  μm, a length of 20 m and is used for homogenization of the beam and shaping a beam with a constant irradiance profile in the plane of the fiber end tip. This beam profile is imaged onto the sample plane using a 12f setup that performs the beam shaping. Therefore, the beam is first collimated with the asphere “ACL3026U-A” (Thorlabs). The two adjustable mechanical slits “VA100/M” (Thorlabs) are located in the image planes of this 12f setup. These slit apertures are used to vary each dimension of the beam profile and, at the same time, the spot geometry in the sample plane. Further, the combination of the two plano convex lenses “LA-1608-A” and “LA1131-A” (Thorlabs) with a focal length of 75 and 50 mm is used to set the beam diameter. Two mirrors “PF10-03-P01” (Thorlabs) propagate the beam upward from the optical table so that the irradiance of the samples inside the container located on the linear axis is possible. In the 12f setup are two more lenses [“LA1509-A” (plano convex) and “LB1676-A” (biconvex) (Thorlabs)] before the beam enters the beam splitter “BS013” (Thorlabs), which splits the beam with a ratio of 50:50. The beam of the optical path propagating to the sample is focused in the sample plane with the high-power objective “LMH-5X-532” (Thorlabs). The other optical path straight ahead through the beamsplitter is detected without focusing with the detector “J-10MB-LE” (Coherent, Inc., Santa Clara, California, United States) for pulse energy measurement. This detector head and the power meter “LabMax-TOP” from the same manufacturer are calibrated in this combination. Since there is no way to measure the pulse energy in-line in the sample plane during the measurement, the pulse energy is determined metrologically in-line with the beam splitter and additionally a characterization of the optical paths is needed to ensure the corresponding value in the sample plane. For this characterization, all optics are inserted, including the optical window “WG12012-A” (Thorlabs) (Fig. 7) to obtain a factor, which describes the ratio of the energy on the sample plane in respect to the measurement plane, by averaging 6 measurements with 2000 pulses for each optical path.

The focusing on the sample is implemented with a moving axis and movement of the container with the sample inside while reviewing the rectangular or square spot geometry of the laser diode on a thin film metal sheeting (thickness: 0.05 mm) located directly on top of the sample. To ensure good results, the laser diode is attenuated with neutral density filters to low irradiance power, not to harm any living cells. The spot is reviewed for focusing with an industrial camera for each sample to cover height differences.

**Fig. 8:**
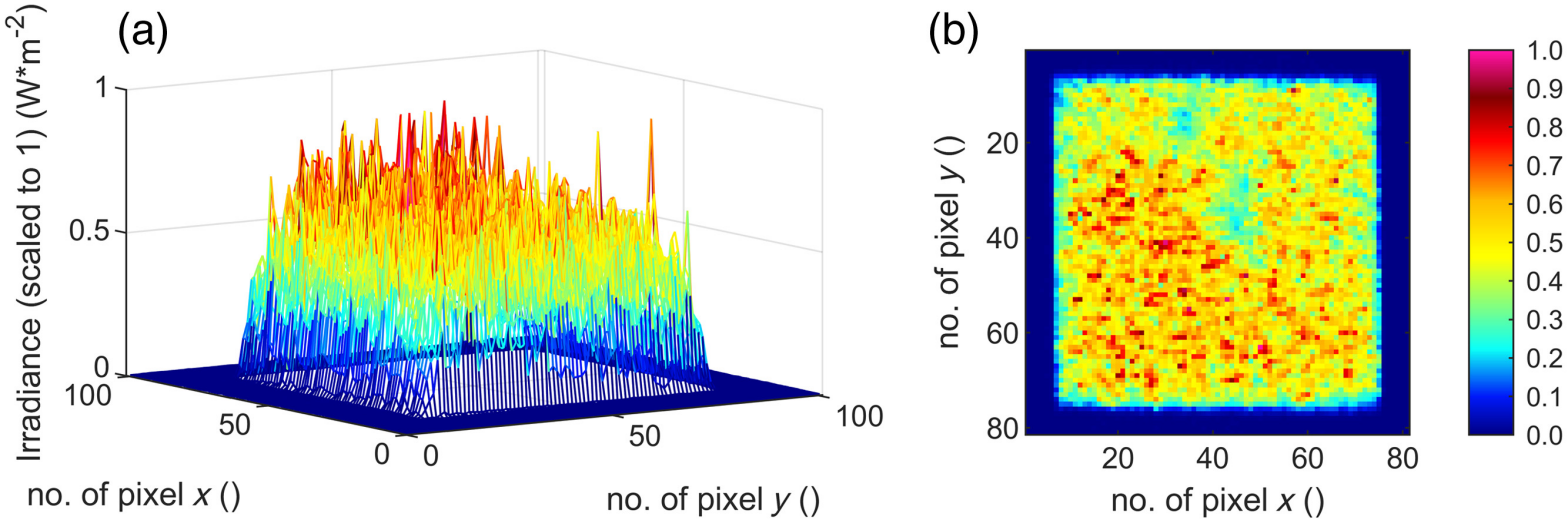
(a) Three- and (b) two-dimensional irradiance plot of spot no. 1 (320  μm×320  μm) in the sample plane.

**Fig. 9:**
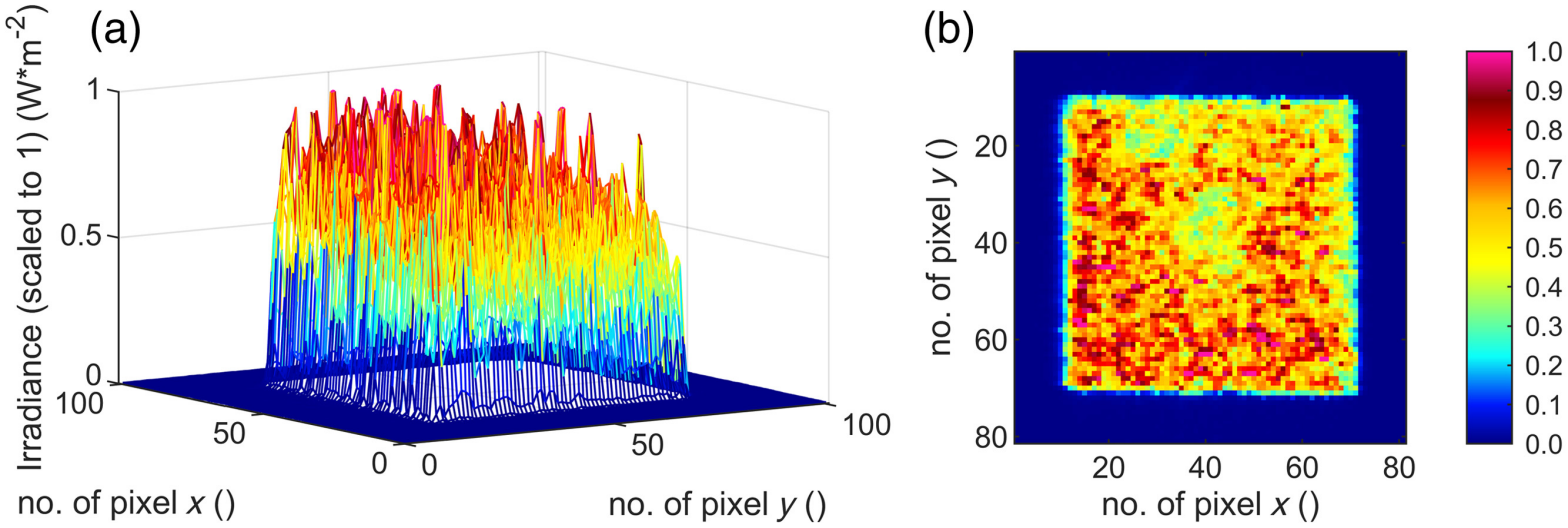
(a) Three- and (b) two-dimensional irradiance plot of spot no. 2 (280  μm×280  μm) in the sample plane.

**Fig. 10:**
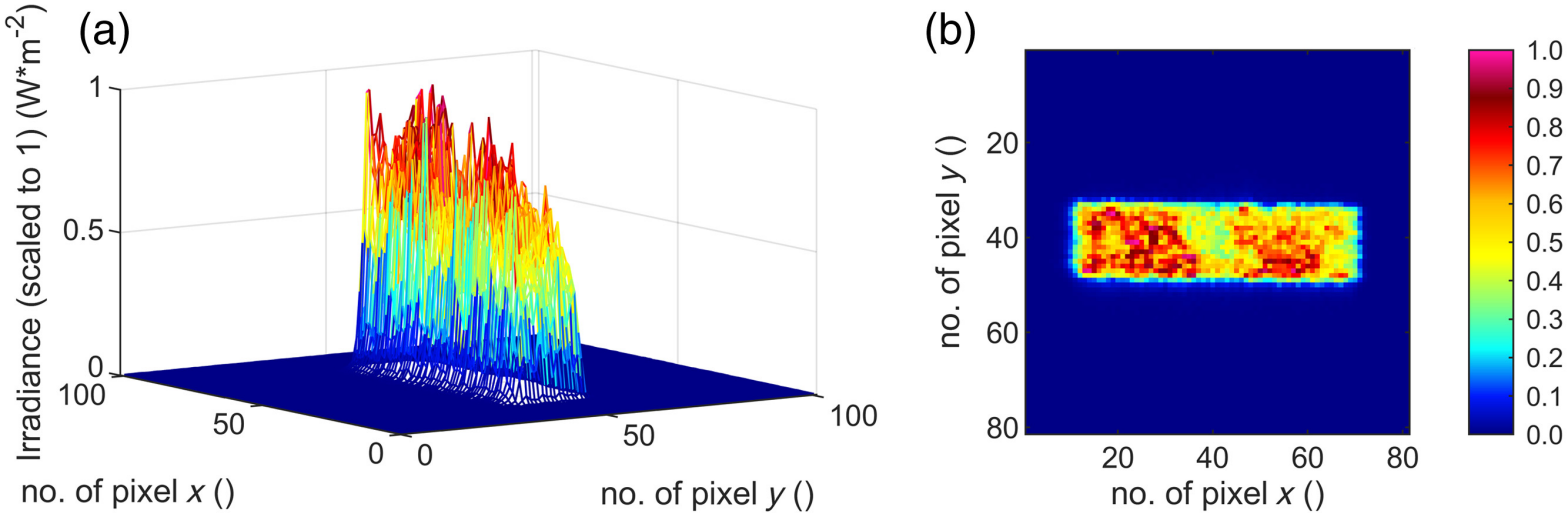
(a) Three- and (b) two-dimensional irradiance plot of spot no. 3 (280  μm×70  μm) in the sample plane.

**Fig. 11:**
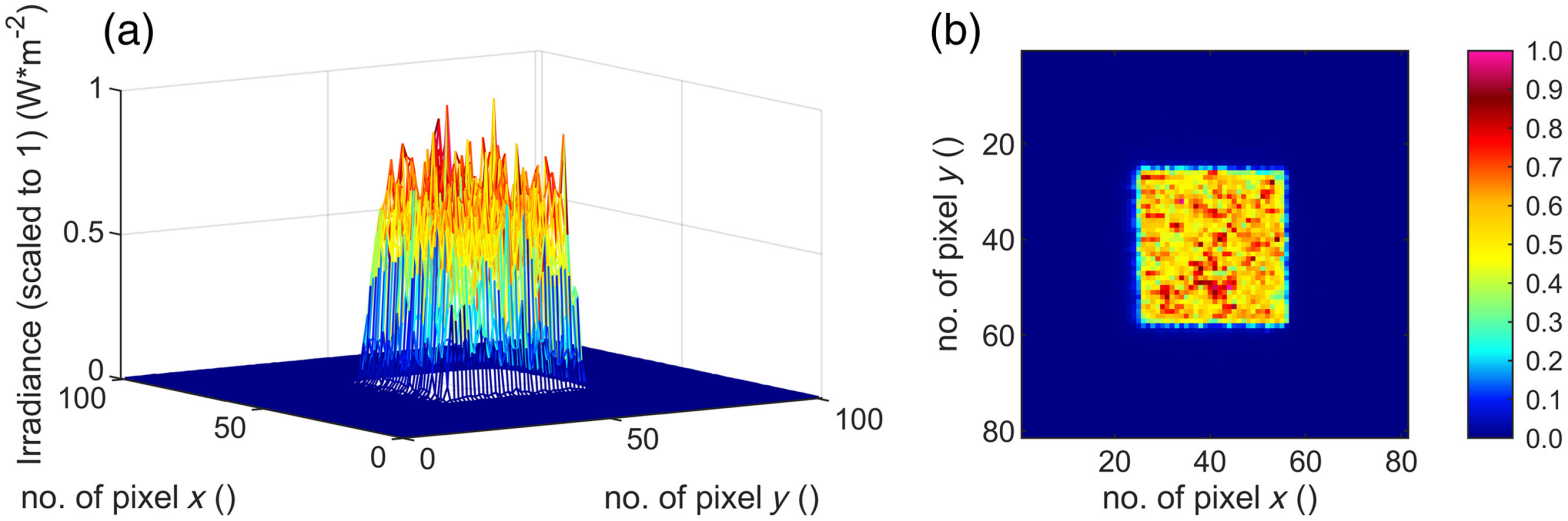
(a) Three- and (b) two-dimensional irradiance plot of spot no. 4 (140  μm×140  μm) in the sample plane.

The irradiation process is done at room temperature of 21°C in an optics laboratory. The resulting four different spot geometries are recorded with the camera “LaserCam-HR II-1/2” (Coherent, Inc., Santa Clara, California, United States) (Figs. 8–11) in the sample plane and listed in Table 3. Twenty single gray scale captures of the laser are taken without going into saturation of the detector and are averaged for the results presented. The spot sizes are calculated with the manufacturer’s information of the pixel size of 4.6  μm.

**Table 3:** Dimensions of the rectangular spot geometries. Spot size no. 3 is targeted to have an aspect ratio of 4:1 to gain information on the elongation. Spot size nos. 3 and 4 share a comparably identical area (targets in parentheses).

No.	Dimension x (μm) (target)	Dimension y (μm) (target)	Aspect ratio	Area (${\mathrm{mm}}^{2}$)
1	317.4 (320)	317.4 (320)	1:1	0.10074
2	282.9 (280)	282.9 (280)	1:1	0.08003
3	282.9 (280)	75.9 (70)	4:1	0.02147
4	144.9 (140)	147.2 (140)	1:1	0.02133

By cutting the spot geometry with slit apertures in the optical setup (Fig. 6), any rectangular spot geometry in the size of 140 to 320  μm can be set up quickly (time ∼5  min) without mechanically changing components. In this work, square and rectangular spot sizes with a ratio of 4:1 (Table 3) are used. Furthermore, the beam profile features a particularly steep flank of the top-hat shape and a strong definition of the irradiated area (Figs. 8–11). A clear spatial separation of the irradiance distribution on the explant tissue is achieved. This in combination with an irradiance profile, which has a constant irradiance distribution, and the application in *ex vivo* explant experiments represents a possibility to generate clearly defined irradiation scenarios. These clearly defined irradiation scenarios can be a sound basis for the laser safety standard—in this case related to porcine explants (for the transfer to humans and NHP see Sec. 5). Especially in comparison with *in vivo* experiments on NHP, where irradiation is performed with a Gaussian beam profile and the size of the retinal spot geometry is calculated^35^^,^^36^ or estimated from the size of the lesion (e.g., $1/e^{2}$ diameter) or specified on the size on the cornea,^37^^–^^40^ it is a significant advantage to be able to determine the exact retinal spot size in the sample position with a detector. In addition, the long-term stability of the optical setup is given and calibrated measurement equipment is used within the *ex vivo* measurements.

#### 3.1.3 Incubation with staining solution

After the irradiation, the viability of the RPE cells of the measurement points has to be examined. Therefore, the samples are prepared for visualization by fluorescence using staining solutions containing calcein acetoxymethylester (calcein-AM) (stock solution contains 1  μg/μl) and propidium iodide (PI) (stock solution contains 1  μg/ml). The calcein-AM assay uses calcein-AM, which is nonfluorescent, passes through cell membranes of living cells and is converted by the cells into green fluorescent calcein. Calcein is used as a marker for living cells, whereas PI marks dead cells, because it only passes through perforated cell membranes of dead cells. The staining solutions are mixed with HBSS in a ratio of 1:200 for calcein and 1:100 for PI and stored in the dark. The samples are placed for 30 min in a 6-well plate in the two staining solutions with an incubation time of 30 min in the dark. Afterward, the samples are washed in HBSS.

#### 3.1.4 Fluorescence microscopy and image analysis

The fluorescence microscope “ApoTome 2” (Carl Zeiss Microscopy GmbH, Jena, Germany) is used to capture z-stack images of the samples with two channels, one for each staining solution. The images captured about 1 h after the irradiation are evaluated by human with the following criteria for the samples and every measurement point, binary information (damage: 1; no damage/not affected: 0) is assigned as done by Schulmeister et al.,^14^ Lipp et al.,^32^^,^^33^ and Schuele et al.^41^^–^^44^

• Samples where the RPE cells detached from the underlying tissue and where the visual check showed unhealthy or abnormal things, such as defects or dryout, are discarded.
• The binary damage criterion for a measurement point has been based on the existence of combined groups of lethal cells in explant damage experiments from the past. A combined group of three or more dead RPE cells next to each other (Ref. 45: 2; Ref. 44: 3; and Refs. 32 and 33: 3) is considered as a lesion. Therefore, the surrounding area of the measurement point or the cells in that area next to the measurement point has to be alive.
• A marker in the shape of an “L” is irradiated with significant higher pulse energy than the measurement points and the expected damage threshold on the tissue [Fig. 13(a)]. Samples where the marker is not visible or did not fulfill the binary damage criterion of three or more dead RPE cells are discarded. The assumption in this case is that the processes of irradiation and staining or handling of the samples mentioned before are not carried out properly or with small errors.
• Areas of the samples where the RPE cells died or had small defects from the preparation are not evaluated, but areas on the same sample with enough living cells around are evaluated.

#### 3.1.5 Statistical evaluation with ProbitFit tool

To process the binary information from the image analysis, a statistical evaluation to determine dose–response data is performed with the ProbitFit Tool by Lund.^46^ The ${\mathrm{ED}}_{50}$ is evaluated and describes the effective dose in which there is a 50% probability of damage. Therefore, all the evaluated measurement points of all the samples for the given exposure scenario are processed with the tool in one evaluation to compensate for statistical fluctuations due to biological variability.

### 3.2 Thermal Simulations

**Fig. 12:**
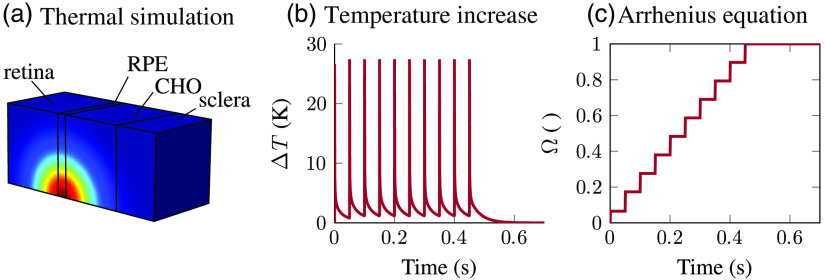
Procedures in the computer model to simulate the retinal thermal injuries of pulsed irradiation patterns. (a) Simulation of the heating in the different tissue layers. (b) Extraction of the temporal temperature behavior of the RPE at the MVL. (c) Insertion of extracted temperature behavior into the Arrhenius equation to simulate the thermal threshold.

In a simulation-based approach, a validated computer model is used to obtain retinal injury thresholds induced by pulsed laser radiation. Such validated models are, however, only available for the retinal thermal damage regime. In this investigation, a computer model based on the work of Jean and Schulmeister,^47^^,^^48^ where a validation was performed with 31 studies including 253 ${\mathrm{ED}}_{50}$ values, is used (simulation parameters and uncertainty; see Ref. 47). In principle, the simulation can be divided into three steps. At first, the irradiance profile on the retina is evaluated using an eye model, which takes the transmittance properties of the anterior eye parts and intraocular scattering effects into account. Then the retinal tissue is simulated and a heat source is defined by the retinal image. Using a finite-element method, the heat transfer equation is solved and the temporal temperature curve is determined for the RPE layer since the retinal injury occurs there first.^49^^,^^50^ In the last step, the temperature along the minimal visible lesion (MVL) is inserted into the Arrhenius equation:^51^^,^^52^

$$\Omega (t)=A\int_{0}^{t}\;\exp(-\frac{E}{RT(t^{\prime })})dt^{\prime },$$ (2)

where R is the ideal gas constant, A is a rate factor with the value $1.05\times 10^{95}\;s^{-1}$, and E is the inactivation energy with the value $5.99\times 10^{5}\;J/\mathrm{mol}$.^47^ A retinal thermal injury is defined where Ω is at least one for all points within the MVL with a diameter of 20  μm.^23^^,^^53^ As a consequence, the criterion where the Ω value equals one at the edge of the MVL is used. Figure 12 shows the model where the heating in the tissue is simulated and where the resulting temporal temperature curve of the RPE is inserted into the Arrhenius equation to simulate a retinal thermal injury.

In this study, nonuniform irradiance profiles with regular pulsed ns-pulses are investigated with regard to *ex vivo* experiments. In order to reflect the *ex vivo* measurement conditions in the computer model, the propagation through the eye model is excluded and the heat source in the retinal layers is defined directly by the nonuniform irradiance profile. To obtain the thresholds for a regular pulsed pattern, the superposition principle is used where the temporal temperature curve in the RPE is simulated for a single pulse and added up afterward according to the pulse pattern.^53^ The simulated retinal thermal injuries in this model are validated for a minimum pulse duration of 100  μs.^47^ For this reason, this single pulse duration is assumed to determine the thermal injuries induced by the background heating of regular pulses in the ns regime.

## 4 Results

### 4.1 *Ex Vivo* Explant Experiments

**Fig. 13:**
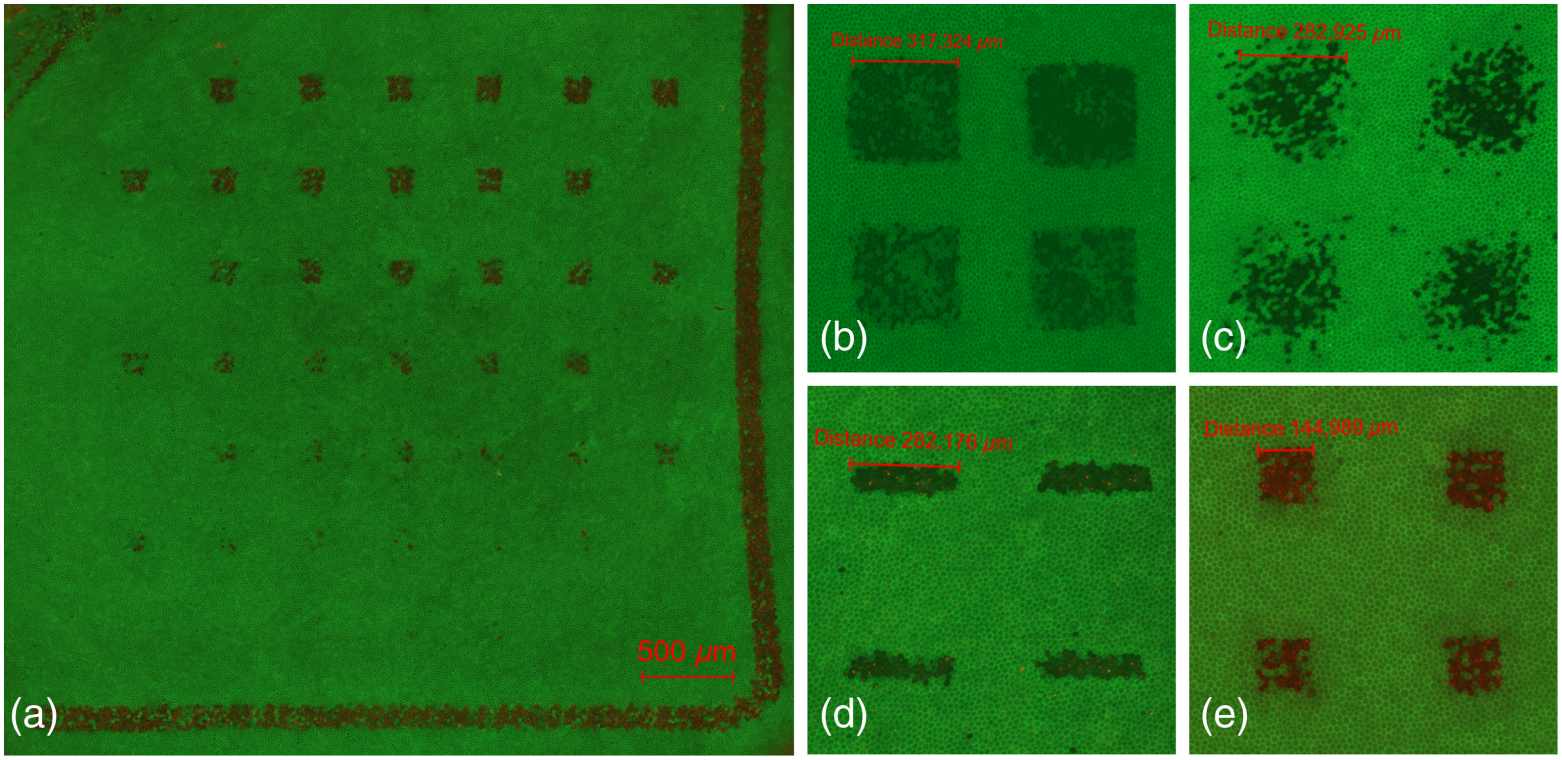
(a) Fluorescence image of a porcine tissue sample (spot size 140×140  μm no. 4) with 42 measurement points in 7 rows. The measurement points with low-pulse energy did not result in a lesion. Close up images for all four spot sizes with high pulse energy: (b) 320×320  μm no. 1; (c) 280×280  μm no. 2; (d) 280×70  μm no. 3; and (e) 140×140  μm no. 4.

The examination of the fluorescence images of the samples shows that the images have clearly defined lesions in the irradiated pattern in terms of damage (Fig. 13). The marker “L” applied for orientation helps to evaluate the process for determining the ${\mathrm{ED}}_{50}$ value (Sec. 3.1), which includes the sample handling, the correct irradiation and the staining of the cells. The elongated shape of the lesions of the retinal spot geometry is distinctly visible on the shape of the lesions, which are sharply defined for the high-energy measurement points. The cells around the measurement points are viable. According to these facts and the combination with the measurement of the spot geometry and shape with a detector in the sample plane, one can be sure that the desired spot geometries are irradiated correctly in focus within the experiments. Focusing is improved by including the metal sheeting on top of the sample.

By varying the pulse energy within the samples in rows [Fig. 13(a)] and between the samples in small steps, measurement points within an interval that includes the ${\mathrm{ED}}_{50}$ value are generated. Thus measurement points with lesions and measurement points without lesions, which are equally desired, can be obtained on each sample. By this, the statistical evaluation is supported with sufficient data to obtain a low slope (defined as ${\mathrm{ED}}_{84}/{\mathrm{ED}}_{50}$ Table 4), which speaks for well-founded measurement data.

**Table 4:** All ${\mathrm{ED}}_{50}$ values from the *ex vivo* measurements in μJ and $\mathrm{mJ}/{\mathrm{cm}}^{2}$ per pulse (spot sizes and scenarios see Tables 2 and 3). The confidence limits (95%) of the statistical analysis are given in parentheses. The corresponding number of measurement days, porcine eyes, and measurement points (M. Points) considered for the calculation are listed. The slope is defined by the fraction of ${\mathrm{ED}}_{84}$ and ${\mathrm{ED}}_{50}$. Parameters: wavelength, 532 nm; pulse duration, 2.2 ns; pulse repetition rate, 20 Hz; and evaluation after 1 h.

Spot size (μm)	Pulses	Days	Eyes	M. Points	${\mathrm{ED}}_{50}$ (μJ)	${\mathrm{ED}}_{50}$ ($\mathrm{mJ}/{\mathrm{cm}}^{2}$)	Slope
320 × 320 (1)	1	2	7	284	38.12 (36.16 to 39.94)	37.84	1.22
320 × 320 (1)	100	1	4	150	32.50 (29.63 to 34.67)	32.26	1.17
280 × 280 (2)	1	2	4	127	42.72 (38.52 to 46.87)	53.38	1.4
280 × 280 (2)	100	3	7	225	35.51 (33.31 to 37.70)	44.37	1.33
280 × 70 (3)	1	2	4	107	10.18 (9.01 to 11.18)	47.41	1.23
280 × 70 (3)	100	1	6	184	10.16 (9.36 to 11.09)	47.31	1.46
280 × 70 (3)	1000	1	4	97	8.46 (7.77 to 9.27)	39.40	1.24
140 × 140 (4)^a^	1^a^	1^a^	6^a^	391^a^	9.30 (9.03 to 9.59)^a^	43.60^a^	1.16^a^
140 × 140 (4)^a^	100^a^	1^a^	6^a^	311^a^	8.32 (8.02 to 8.61)^a^	39.01^a^	1.15^a^

a Measured with 5 m fiber length (all other data with 20 m).

**Fig. 14:**
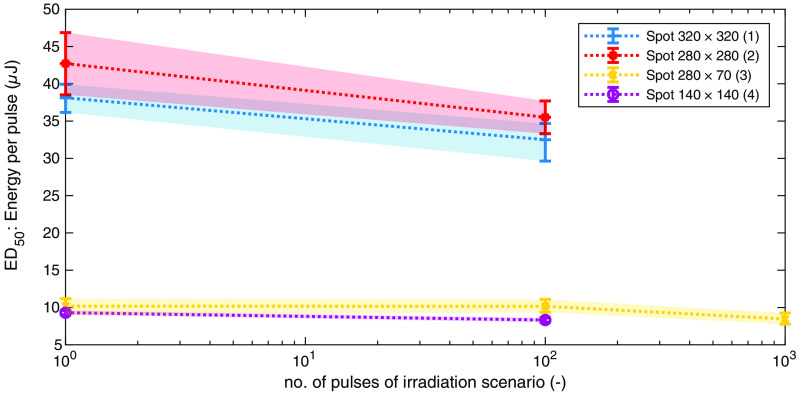
Overview of all ${\mathrm{ED}}_{50}$ values in μJ of the explant experiments for all four spot sizes and no. of pulses (Tables 2 and 3). The confidence limits (95%) of the statistical analysis are illustrated with errorbars and colored surfaces.

The process of sample handling, irradiation, staining, fluorescence evaluation, and statistical analysis introduced by Lipp et al.^32^^,^^33^ proved successful. Afterward, a large proportion of the samples showed living, green-stained cells at the desired locations. The results (dose–response data) for all series of measurement and all spot sizes are listed in Table 4 and shown in Fig. 14 with the corresponding upper and lower limits (confidence intervals).

The overall two ${\mathrm{ED}}_{50}$ values for the spot size 320×320  μm no. 1 are lower than the corresponding values for the spot size 280×280  μm no. 2, although the irradiated area is about 1.259 times larger (Table 3). The reasons for this may lay in the variation of the handling of the samples, general biological variability of the tissue and focusing. In the case of focusing, it occurred that the spherically shaped tissue samples tend to bulge during insertion and clamping inside the sample holders, if the handling was not correct. It is therefore advantageous to irradiate the samples directly after clamping them in the sample holders, because in some cases bulging has occurred over time and has also increased over time.

For all spot sizes, the results show a reduction of the ${\mathrm{ED}}_{50}$ values with increasing number of applied pulses (Fig. 14). The reduction in case of the spot size with the rectangular geometry (280×70  μm no. 3) from 1 to 100 pulses is the smallest (10.18 to 10.16).

The significance of our measurement results can be evaluated on the basis of the slope, which appears to be sufficient to very good with values between 1.15 and 1.46. Since at least 4 different eyes and at least 97 evaluated measurement points with surrounding living cells (see criteria in Sec. 3.1.4) are consulted for the statistical evaluation for each ${\mathrm{ED}}_{50}$ value, the results have been obtained on the basis to a sufficient extent.

### 4.2 Thermal Simulations

**Fig. 15:**
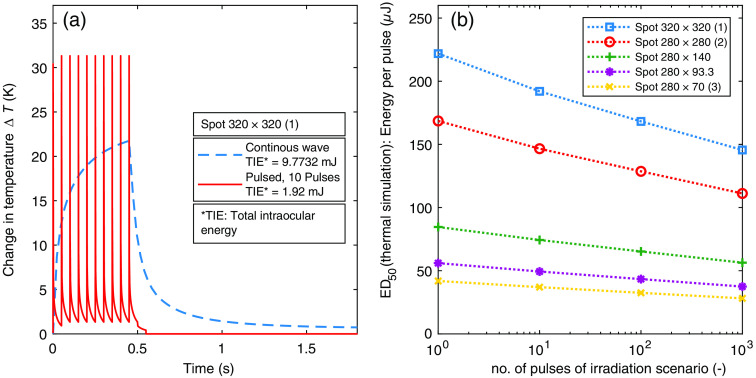
(a) Simulated temporal temperature curve of the RPE layer with a CW (blue) and pulsed (red) irradiation scenario for the spot size 320×320  μm no. 1. (b) Simulated ${\mathrm{ED}}_{50}$ values for all spot sizes (Table 5).

To simulate thermal damage in pulsed irradiation scenarios, the temperature profile of a single pulse is simulated initially and a pulsed scenario is received subsequently using the superposition principle (Sec. 3.2). The result is a simulated temporal temperature curve of the RPE tissue layer [Fig. 15(a)], where the change of temperature is plotted versus the irradiation time. Figure 15(a) shows two irradiation scenarios, the main pulsed one studied for this work and a comparable one with continuous wave (CW) irradiation with the same total duration. It is evident that the simulated background heating of the tissue by short pulses (100  μs) is higher for a short time than for CW irradiation but decreases fast between the pulses. In the case of CW irradiation, the temperature increases continuously. In general, stronger background heating, even for a short time, results in a higher probability of thermal damage, which is determined from the temperature curve using the Arrhenius equation [Eq. (2)]. The results with pulsed scenarios therefore yield lower ${\mathrm{ED}}_{50}$ values than simulations of CW irradiation and the total intraocular energy TIE is lower as well. This is why a potential risk of thermal damage is much higher in pulsed scenarios, where denaturation processes may also lead to a thermal damage.

All ${\mathrm{ED}}_{50}$ values per pulse calculated from the temperature curves of pulsed scenarios are shown in Table 5 and Fig. 15(b) depending on the irradiation scenario with a certain aspect ratio. The ${\mathrm{ED}}_{50}$ values decrease with an increase in the number of pulses for all spot sizes as well as the elongated spot geometries for the thermal damage simulations. This is because a lower energy of the individual pulses is required to produce a thermal damage if the number of pulses increases.

**Table 5:** Overview about the simulated thermal ${\mathrm{ED}}_{50}$ values per pulse for different spot sizes with different aspect ratios. Simulation parameters: wavelength: 532 nm; pulse duration: 100  μs; pulse repetition rate: 20 Hz.

Spot size (μm)	Aspect ratio	Area (${\mathrm{mm}}^{2}$)	Simulated ${\mathrm{ED}}_{50}$ per pulse (μJ)			
			1 pulse	10 pulses	100 pulses	1000 pulses
320 × 320 (1)	1:1	0.1024	221.82	192.00	168.20	145.59
280 × 280 (2)	1:1	0.0784	168.54	146.57	128.66	111.14
280 × 140	1:2	0.0392	84.61	74.33	65.24	56.36
280 × 93.3	1:3	0.0261	56.02	49.37	43.37	37.46
280 × 70 (3)	1:4	0.0196	41.89	36.99	32.49	28.13

**Fig. 16:**
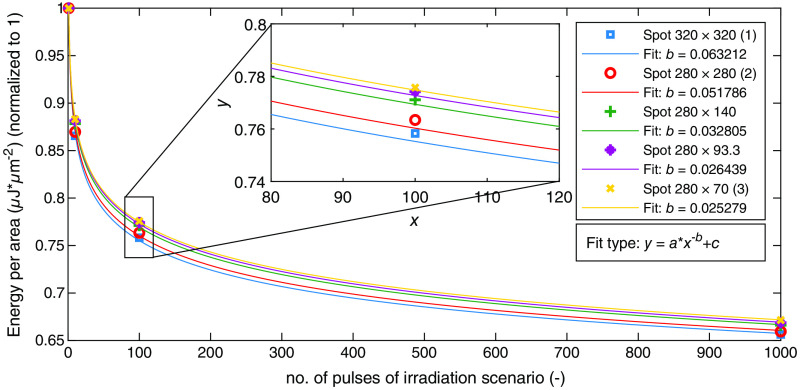
Simulated ${\mathrm{ED}}_{50}$ values expressed as irradiance for the spot sizes normalized to 1. Additional fits with the parameter b for evaluation in the legend of the plot.

In order to investigate the effect of elongation of the selected spot geometries, the ${\mathrm{ED}}_{50}$ values are considered in relation to the area of the respective spot size and normalized to 1, which is shown in Fig. 16. Both the behavior of the decay with increasing number of pulses and the magnitude of the ${\mathrm{ED}}_{50}$ values (y axis value) is the same. Dependencies with respect to thermal damage can be identified reviewing the fit parameters of the power fit applied (see parameter b in Fig. 16). The parameter b decreases with decreasing area of the spot size and with increasing elongation. All the data points show these dependencies. For thermal damage, these data indicate a minimal dependence of the damage threshold on one hand on the area of the spot geometry or on the other hand on the elongation or both contributing factors together. With both contributing factors, it is very likely that this can result due to the geometry and the heat flow inside the tissue, as these are the only major differences.

For thermal simulations, a pulse duration of 100  μs is selected instead of the actual pulse duration in the nanosecond time regime of ∼2.2  ns, because a simulation with the validated model, however, is no longer purposeful with pulse durations below 100  μs. For durations <100  μs, the thermal relaxation time is undercut and the background heating of the tissue by thermal conduction can physically no longer be modeled correctly or is no longer achieved in reality. Furthermore, for durations <100  μs, inhomogeneous absorption properties are present inside the tissue and a local temperature increase is very inhomogeneous. In the nanosecond time regime, the thermomechanical damage mechanism based on the formation of microbubbles on the melanosomes of the RPE cell layer dominates.^43^^,^^54^^–^^56^ However, it is of interest for the understanding of retinal damage mechanisms and the derivation of further results by comparing the simulation and measurement data.

### 4.3 Combined Results

**Fig. 17:**
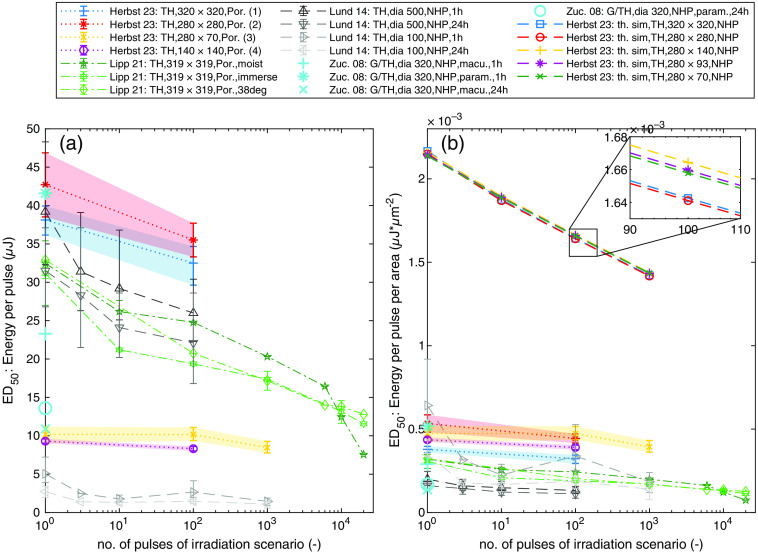
(a) Comparison of the experimentally determined ${\mathrm{ED}}_{50}$ values in μJ per pulse with literature sources (532 nm, lower nanosecond time regime). The confidence limits (95%) of the statistical analysis are illustrated with errorbars. (b) Comparison of the experimentally and computationally determined values in μJ per pulse and per area with the experimental literature sources. Sources: Refs. 32, 33, 36, and 57 (TH, top-hat; G, Gauss; 320×320, rectangular spot geometry 320 to 320  μm; dia 500, diameter of spot geometry of 500  μm; Por., porcine *ex vivo* measurements; NHP, NHP *in vivo* measurements; macu., macula; param., paramacula; 1 h, evaluation time 1 h; and th. sim., thermal simulations).

For comparison of the damage thresholds, the ${\mathrm{ED}}_{50}$ values of the *ex vivo* measurements are plotted in Fig. 17(a) against literature sources, which represent parts of the data basis of the laser safety standard. A comparison is made with literature sources with a wavelength of 532 nm with pulse durations in the lower nanosecond time regime and with NHP or porcine tissue. In addition, in Fig. 17(b), all data sources are divided by the respective measurements’ retinal image size for comparability. However, it should still be clearly noted that for this comparison the parameters, such as the animal tissue, the pulse duration, repetition rate, and the type of measurements, widely differ between the literature sources. In addition, the simulation results are plotted in Fig. 17(b). The measured ${\mathrm{ED}}_{50}$ values from this work seem to be at the upper end of the available data sources, which can be seen in Fig. 17(b).

The best matching literature source that corresponds to this work in terms of measurement parameters and type of animal samples is the series of measurements by Lipp et al.^32^^,^^33^ (immersed porcine explants, spot size 319  μm×319  μm). In the case of single pulses, the ${\mathrm{ED}}_{50}$ value of this work is 38.12  μJ or $37.84\;\mathrm{mJ}/{\mathrm{cm}}^{2}$ (spot size 320×320  μm no. 1) per pulse compared to 31.16  μJ or $30.62\;\mathrm{mJ}/{\mathrm{cm}}^{2}$ (Ref. 33), which is in the same order of magnitude and differs by a factor of ∼1.223 or 1.236. Comparable slopes of the measurement series of this work [dashed straight lines in Fig. 17(a)] for increasing number of pulses with literature sources are apparent for the larger spot sizes [spot size 320×320  μm no. 1, spot size 280×280  μm no. 2, Lipp et al.^33^ (spot size 319×319  μm), Lund et al.^57^ (diameter of 500  μm)] and for smaller spot sizes (spot size 280×70  μm no. 3, spot size 140×140  μm no. 4, Lund et al.^57^ [diameter of 100  μm)]. In summary, it can be said that the differences within the measurement results including all literature sources are quite high. The evaluation of Zuclich et al.^36^ shows that the ${\mathrm{ED}}_{50}$ can differ by a factor of 4 purely due to differences in the “postexposure read time”^36^ and the location of irradiation (macula versus paramacula), which is enormous. Between the samples of one irradiation scenario or spot size, it is noted that the threshold values sometimes strongly differed between the samples. This is why the statistical evaluation of all measurement points for one irradiation scenario is done in one dose–response curve to calculate an ${\mathrm{ED}}_{50}$ value. These differences between the samples of our experiments are larger than the differences determined between elongated spot geometries (spot size 280×70  μm no. 3) compared to nonelongated spot geometries with the same area (spot size 140×140  μm no. 4). At present, it is therefore not possible to say whether elongated spot geometries behave differently with respect to the ${\mathrm{ED}}_{50}$ value than comparable spot geometries with the same irradiation area in *ex vivo* measurements.

The most important result of the *ex vivo* measurement series is that the ${\mathrm{ED}}_{50}$ values are not resolved finely enough to determine an effect relying on the elongation of the spot size. Concluding that, three major influencing factors can be identified from our measurements that significantly affect the accuracy of *ex vivo* damage threshold measurements, which are the biological variability of the animal tissue, the focusing on the samples and the evaluation methods and damage criteria. Many literature sources do not address the latter factor, but it matters hugely if the sample that has no lesion at the marker and requires about 50% to 100% more energy to have a lesion is included into the evaluation or categorized as unusable.

In the following, the assumption is made that *ex vivo* explant measurements behave in the same way as thermal simulations (NHP) including the validated damage model with respect to increasing number of pulses. Based on the value level and the significantly steeper sloping curves of the simulated ${\mathrm{ED}}_{50}$ values in the Fig. 17(b), it can be concluded under this assumption that the *ex vivo* measurements in this work are not based on a thermal damage mechanism.

## 5 Discussion

Based on the three major influencing factors, that significantly affect the accuracy of *ex-vivo* damage threshold measurements, presented above and the substantial number of damage threshold experiments performed individually, we see a need for a standardized or at least common practice for damage threshold experiments. This is the best way to establish meaningful comparability between the different measuring methods and parameter sets. A common practice is necessary to precisely define retinal damage thresholds, since a difficulty of assessing the presence of a damage exists. The way of determining a lesion, whether this is within *in vivo* experiments on NHP or within *ex vivo* experiments, has been and continues to be important. A common practice can lead to more clarity, but the underlying criterion must also be based on a definitive basis for decision-making. For this purpose, a combination of a calcein and PI assay is used in this work, but in the future, other types of markers or assays could certainly be suitable and explored.

There are many influencing parameters besides the five major parameters (wavelength, pulse duration, spot size, pulse repetition frequency, and number of pulses) to the ${\mathrm{ED}}_{50}$ value. For example, for the assessment time after irradiation, the value of 24 h has become the standard, as it corresponds to a lower energy value and more likely equals the worst case, thus establishing a common practice for this parameter. One goal is to determine the accuracy of the damage experiments in regard to the influencing factors and develop reasonable procedures for future damage threshold experiments to minimize these factors.

Between the samples of our series of measurements, it was noted that the threshold values sometimes strongly differed between each sample. The determination of biological variability in the measurement of damage thresholds should therefore be aimed at. There is a need for additional measurement series that include fewer influencing factors for the same irradiation scenario [e.g., temperature of the samples, location of the irradiation (macula versus paramacula)].

Another issue is the presence of speckle or peaks of the irradiance in the sample plane, which are present in the measurement setup of this work. This issue was addressed by the use of correction factors in the previous literature sources (e.g., “speckle factor”^58^ or “intensity modulation factor”^33^^,^^44^). A standardized or common practice should be used to correct spatial beam profiles since the measurement of such a correction factor depends massively on the detector used and its spatial resolution. This is why no correction of the ${\mathrm{ED}}_{50}$ values is applied in this work. Further studies are proposed to develop methods that adjust and correct different irradiance distributions and beam profiles (top-hat/Gaussian) with respect to retinal damage thresholds.

As this work presents initial damage thresholds for rectangular irradiation scenarios obtained in experiments, an explicit proposal is made to expand this database to understand whether the different behavior in terms of the temperature gradient shown in Fig. 3(b) plays a role for the damage threshold. The first reason is to understand damage mechanisms in the retinal tissue, in order to be able to set up and validate models and simulations of the thermomechanical damage mechanism. A common practice could include procedures with markers that help to evaluate if microbubbles or mechanical shock forces were present and therefore help to understand laser-induced retinal damage mechanisms. The second reason is that it is important for the manufacturers to ensure eye safety and optimize the energy emitted by their products.

### 5.1 Advantages of *Ex Vivo* Explant Experiments

To further develop the possibilities given by *ex vivo* explant measurements, there is a need to optimize the measurement method itself and the comparability by generating more accurate data for the laser safety standard or the occupational safety in general. Schulmeister et al.^14^ stated that a “precise dosimetry of the energy incident as well as the beam profile is possible” with explant measurements and that “stable samples and no influence of corneal clouding or aberrations”^14^ are given. Our research adds that a reasonably good definition of the spot geometries can be achieved using similar optical setups as in our setup. Further, the dimensions of the irradiated area on the retinal tissue can be measured directly. Performing *ex vivo* measurements, the exact beam shape or retinal image applied to the tissue and the parameters of the pulses on the sample plane are known exactly within the measurement tolerances. Therefore, results of *ex vivo* measurements can be compared best to the results of simulations. Sliney et al. stated that “observer skills and lesion detection”^5^ influence the ${\mathrm{ED}}_{50}$ values within *in vivo* measurements. Since *ex vivo* explant experiments are able to deliver samples with binary information on the liveliness of cells via fluorescence microscope images and the criterion of damage depends on the same information, this method has the best opportunity to be automated by scripts or image evaluation removing the human factor and the best possible determination of damage thresholds.

### 5.2 *Ex Vivo* Explant Experiments Results’ Impact on the Laser Safety Standard

A disadvantage of *ex vivo* explant measurements is that for the interpretation of the *ex vivo* measurement results and the usage of these results in the laser safety standard still the transfer to the human eye is needed. On one hand, the transfer from porcine tissue to human tissue needs to be investigated for example on the behalf of anatomical and intra- and extra-cellular structure of the tissue samples, even though the similarities of these two species are high. On the other hand, the transfer from *ex vivo* explant tissue experiments to *in vivo* experiments of other species (e.g., NHP) needs to be investigated.

## Data Availability

All data in support of the findings of this paper are available within.
